# Metal–Hydrogen Batteries and Decoupled Electrolyzers: Converging Pathways for Hydrogen-Integrated Energy Storage

**DOI:** 10.1021/acsenergylett.5c03575

**Published:** 2026-03-27

**Authors:** Laura Sanchez-Cupido, Begoña Acebedo, Nagore Ortiz-Vitoriano, Paramaconi Rodriguez

**Affiliations:** † Center for Cooperative Research on Alternative Energies (CIC energiGUNE), Basque Research and Technology Alliance (BRTA), Alava Technology Park, Albert Einstein 48, 01510 Vitoria-Gasteiz, Spain; ‡ Ikerbasque, Basque Foundation for Science, María Díaz de Haro 3, 48013 Bilbao, Spain

## Abstract

Electrochemical energy storage and hydrogen conversion have historically been developed as separate technologies, yet their chemistries and engineering challenges overlap profoundly. Metal–hydrogen (M–H_2_) batteries and redox-mediated, membrane-free electrolyzers both rely on reversible hydrogen redox chemistry in aqueous or hybrid electrolytes. This Perspective examines how these systems, once considered distinct, are converging at the level of redox chemistry, materials challenges, and benchmarking needs while retaining distinct architectures and engineering constraints for dispatchable, long-duration energy storage. It summarizes benchmark metrics for each platform, identifies shared scientific foundations, and outlines design principles for mediators, electrodes, and interfaces. Emphasis is placed on best practices for standardized testing and reporting, cross-disciplinary benchmarking, and near-term research directions. By viewing M–H_2_ batteries and decoupled electrolyzers as complementary elements rather than competitors, it becomes possible to envision a cohesive hydrogen-integrated energy infrastructure spanning seconds-to-seasons storage.

## Graphical abstract

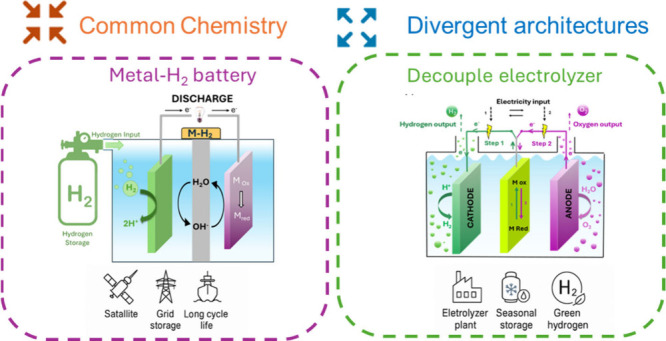

## M–H_2_ Batteries and Decoupled Electrolyzers: From Parallel Evolution to Convergence

Over the past decade, the rapid growth of global electrification has far exceeded the rate of progress in grid flexibility. Rapidly varying renewable generation demands require technologies capable of fulfilling both fast response and long-duration buffering. Conventional batteries deliver high round-trip efficiency over short time scales, whereas H_2_ provides essentially unlimited duration and transportability.^1–4^ Between these domains, M–H_2_ batteries and decoupled electrolyzers have emerged as two ends of a shared electrochemical ground.

A M–H_2_ battery is a hermetically sealed system, where no fresh electrolytes are supplied and the H_2_ formed is stored and then reoxidized during discharge to regenerate electrical energy. Because the H_2_ never leaves the device, it serves purely as an internal redox carrier, enabling thousands of reversible cycles with no net mass exchange (Figure 1). To confine the gas while maintaining ionic conduction, a H_2_-permeable yet electrolyte-impermeable separator (typically a Pd-alloy foil) is indispensable, and the electrodes are permanently wired inside a rigid enclosure, which limits geometric flexibility and scale-up routes.^5–8^ M–H_2_ batteries hinge on efficient H_2_ storage, for which several physical and chemical strategies have been developed.^9,10^ Classic Ni–H_2_ batteries, such as those used in spacecraft, rely on a pressurized alkaline electrolyte and are known for their remarkable longevity, offering thousands of deep cycles with minimal degradation.^11–13^ More recent developments in M–H_2_ chemistry, such as Mn–H_2_ ^14,15^ and Li–H_2_ ^6^ systems, have expanded the voltage range and offer even greater energy storage capacity. These batteries are valued for their reliability, the use of nontoxic materials, and their ability to quickly respond to power demands, making them ideal for applications that require long-term stability and rapid energy release.

**Figure 1:**
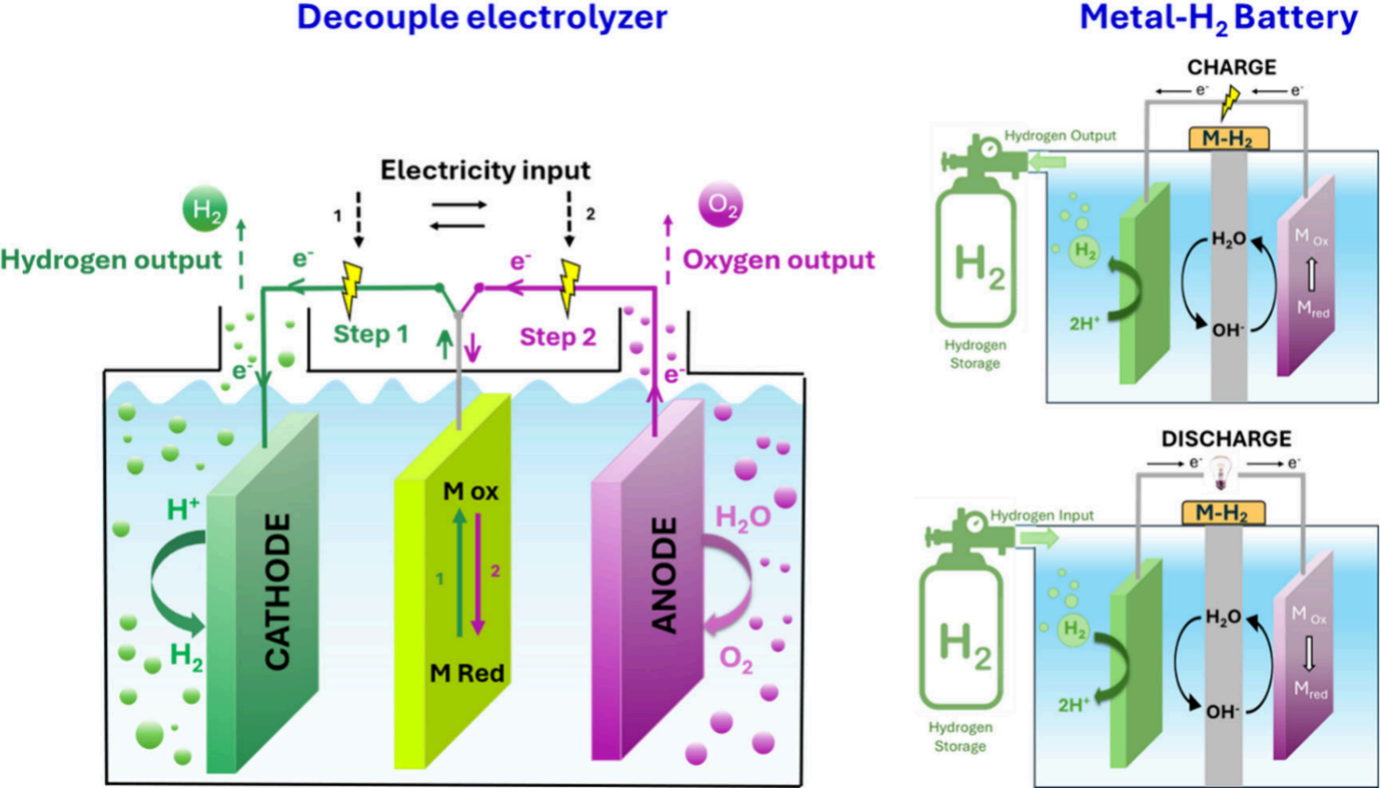
Scheme with concept and mechanisms of a decouple electrolyzer and the charge and discharge process of a metal–H_2_ battery.

Redox-mediated or decoupled electrolyzers approach the H_2_ cycle from a fundamentally different perspective than traditional electrolyzers. Instead of keeping the oxygen evolution reaction (OER) and hydrogen evolution reaction (HER) in the same electrochemical cell, redox-mediated electrolyzers separate these two reactions in time or space (Figure 1). They operate as open, flow-through reactors in which liquid water—or an alkaline or acidic supporting electrolyte—is continuously fed, while H_2_ and O_2_ are withdrawn immediately after evolution.^16,17^ This prevents accumulation and allows steady-state production rates to be controlled by the feed flow rather than by the state-of-charge. This separation is achieved through the use of a reversible redox mediator, which can be either solid or soluble.^16,18–21^ Common redox mediators include Ni­(OH)_2_/NiOOH,^17,18,22,23^ MnO_2_/MnOOH,^24,25^ and Bi/Bi_2_O_3_.^26^

The key innovation in redox-mediated electrolyzers is their ability to eliminate the need for polymer membranes, which are traditionally used in proton-exchange membrane (PEM) and anion-exchange membrane (AEM) electrolyzers to prevent the mixing of H_2_ and O_2_ gases. This membrane-free design not only simplifies the system but also reduces costs associated with membrane degradation and complex gas management.^27,28^ The result is intrinsically safe, high-purity H_2_ generation that can operate efficiently under intermittent renewable power.

In order to clarify the differences, Figure 2 schematically compares the technologies of M–H_2_ batteries and redox-mediated (decoupled) electrolyzers, highlighting their difference in reactor openness, functional objective, H_2_ handling, and cell architecture.

**Figure 2:**
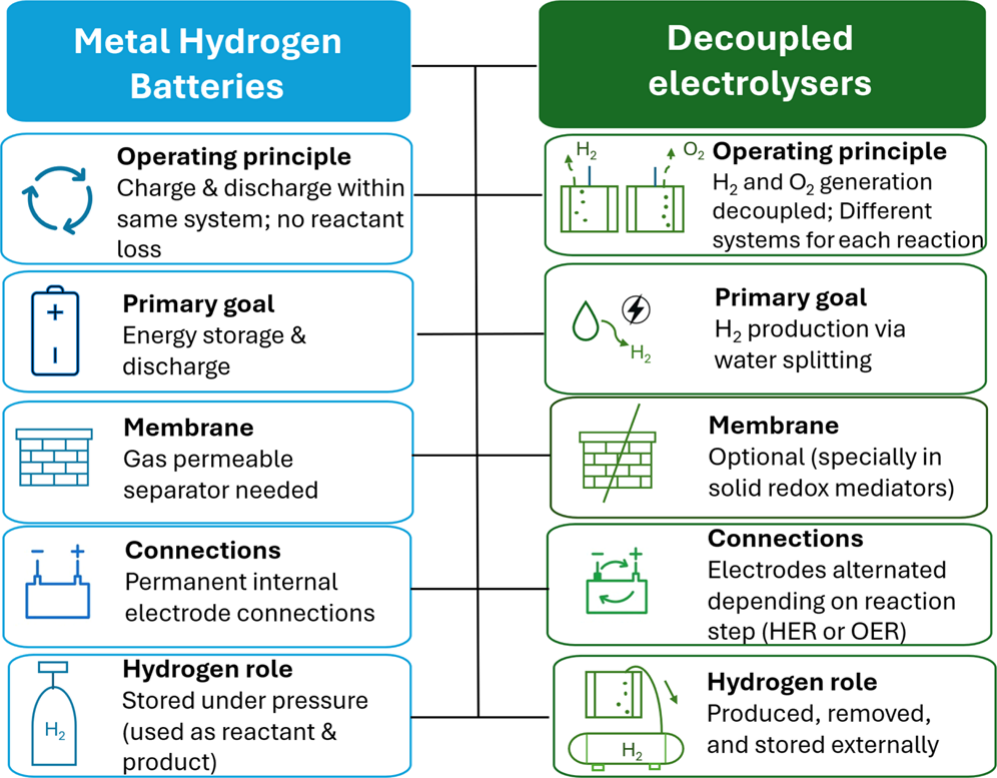
Comparative diagram of both technologies: M–H_2_ batteries and solid-based redox mediators decoupled electrolyzers.

Viewed together, these devices reveal a meaningful overlap in redox chemistry and materials challenges despite fundamental differences in system architecture, hydrogen management, and operating objectives. Both exploit proton-coupled electron transfer in similar potential ranges, rely on layered hydroxide or oxide frameworks, and share durability and scaling constraints while differing in operating principle, hydrogen management, and engineering aspects. The aim of this Perspective is not to retrace the separate histories of these two families but to clarify where they converge and diverge scientifically and to show how coordinated research efforts can accelerate progress in both.

## Shared Redox Foundations

The conceptual border between a *battery electrode* and a *mediator plate* therefore becomes semantic. Both are solid hosts mediating proton-coupled electron transfer with H_2_ as the ultimate counter-species. Recognizing this equivalence is the intellectual foundation for cross-disciplinary benchmarking. To exemplify, currently at the heart of both technologies lies the reversible Ni­(OH)_2_/NiOOH transformation. In Ni–H_2_ batteries, this couple serves as the positive electrode: Ni­(OH)_2_ is oxidized to NiOOH during charge while H_2_ evolves at the negative electrode; discharge reverses the reaction, consuming stored H_2_ internally.^7,29^ In a decoupled electrolyzer, the same couple functions as a solid mediator, oxidized during the HER step and reduced during OER, thereby temporally separating the two half-reactions.^17,18,22,23^ This commonality extends to reaction intermediates, lattice breathing, and degradation modes. Both experience α-/β-/γ-phase transitions, conductive-path loss upon prolonged cycling, and carbonate contamination under ambient CO_2_. These shared mechanisms imply that advances in one field directly inform the other: improved dopants or coatings that stabilize NiOOH in battery cycling will likewise extend mediator lifetime in decoupled electrolysis; that is, decoupled electrolyzers can draw from the considerable experience accumulated in NiMH and Ni–H_2_ systems in areas such as durability testing, component degradation, and performance characterization. At the same time, next-generation M–H_2_ batteries can take advantage of the insights gained from the decoupled-electrolyzer community, especially those related to system integration, balance-of-plant considerations, and pilot-scale operations.

A good example of a battery development with a Mn-based cathode is demonstrated by Chen et al.,^14^ where the system operates via a redox reaction between Mn^2+^ and MnO_2_ at the cathode, coupled with hydrogen evolution and oxidation reactions (HER/HOR) at the anode. This Mn-based battery boasts several advantages, including a discharge voltage of approximately 1.3 V vs the standard hydrogen electrode (SHE), fast charge/discharge capabilities (up to 100 mA cm^–2^), and a lifetime of over 10000 cycles without degradation. With an energy density of around 139 Wh kg^–1^ and the use of low-cost materials, this system presents a promising solution for renewable energy storage. Similarly, in the field of decoupled electrolysis, MnO_2_/MnOOH has been successfully employed as a redox mediator, offering similar advantages of fast charge/discharge kinetics, high efficiency, and long cycle stability.^24,25^ These Mn-based systems, whether in a battery or decoupled electrolysis mode, show great promise due to their combination of low-cost materials and high performance under varied conditions. In a different application, LiMn_2_O_4_ (spinels), demonstrated by Zhu et al.,^15^ has been shown to be an efficient anode material for Mn–H_2_ batteries, achieving a specific capacity of 83 mAh g^–1^ at 1 C and retaining 69.1 mAh g^–1^ even at ultrahigh rates (50 C). These results underscore the potential of Mn-based systems to deliver a favorable blend of energy density, fast charge capabilities, and cycle stability—key attributes that make them viable for grid-scale energy storage applications.

Xu et al.^26^ introduced Bi_2_O_3_ as a novel mediator for decoupled electrolysis, not used so far for M–H_2_ batteries, enabling near-100% Faradaic efficiency. The Bi_2_O_3_/Bi redox cycle facilitated both HER/OER with an overall cell potential of 0.45 V for HER and 1.19 V for OER. The system demonstrated 300.8 mAh g^–1^ reversible capacity with excellent cycle retention. The authors also explored using Zn oxidation to Zn­(OH)_2_ as an alternative to the oxygen production reaction, thus integrating H_2_ production and a Bi_2_O_3_–Zn battery, showing stable cycling and good performance over thousands of cycles. In the same direction, Hahn et al.,^30^ developed the Zn–H_2_ energy storage system, operating similarly to a decoupled electrolyzer using the Zn/ZnO redox couple. It produces electricity and H_2_ simultaneously during discharge without external energy input and uses low-cost Ni alloy catalysts for HER and OER. Despite catalyst degradation over long cycles, the system offers high efficiency and robust cycling performance.

Operando characterization provides the critical link for unifying understanding between M–H_2_ batteries and decoupled electrolyzers. Synchrotron X-ray and neutron diffraction allow for real-time tracking of structural evolution, revealing phase transitions, lattice strain, and the growth of degradation products under operational conditions.^31^ X-ray absorption spectroscopy (XAS) is particularly useful for mapping oxidation-state dynamics of key redox-active materials, such as Ni­(OH)_2_/NiOOH, MnO_2_/MnOOH, and Zn/Zn­(OH)_2_ as they cycle between their oxidized and reduced forms. This helps to identify the stability of the materials and potential for phase separation or dissolution during cycling. Solid-state nuclear magnetic resonance spectroscopy (NMR) provides detailed insights into proton transport within the electrolyte and the surface speciation of hydroxide ions, shedding light on ion diffusion mechanisms and interfacial behavior,^32,33^ crucial for understanding charge transfer in both M–H_2_ systems and electrolyzers. Raman spectroscopy further complements these techniques by offering high-resolution, nondestructive analysis of vibrational modes in electrodes, capturing the evolution of functional groups and any formation of reaction intermediates.^34^ By employing these common diagnostic protocols across both technologies, we can create unified data repositories that promote collaboration, allowing degradation models, kinetic parameters, and mechanistic insights to be shared between battery and electrolyzer communities, accelerating advancements in both fields.

*The real pivot is architectural: common mediators, common interfaces, shared electrochemical components, optimize once, leverage across both devices.*

## Current Landscape and Benchmark Metrics

Performance comparisons across the two fields have been hampered by inconsistent metrics. A unified perspective begins by framing a few quantitative metrics. M–H_2_ batteries typically operate at voltages around 1.2–1.3 V for Ni- or Mn-based systems^14,29,35,36^ and approximately 3 V for Li–H_2_ cells.^6^ These batteries are known for their remarkable round-trip efficiencies of 60–85%, and demonstrated lifetimes exceeding 10000–30000 cycles,^8^ provided that pressure-vessel constraints are properly managed. In some designs, H_2_ is stored in the form of metal–metal hydrides, which offer high volumetric energy densities and enhance the safety and stability of H_2_ storage. These alloys, such as lanthanum–nickel (LaNi_5_) or other intermetallic compounds, store H_2_ at moderate pressures, making them reliable storage media for long-duration applications. Energy densities near 150 Wh kg^–1^ are typical for Ni–H_2_ and Mn–H_2_ systems,^14,29,37^ while newer Li–H_2_ batteries approach higher energy densities, with some prototypes reaching closer to 700–800 Wh kg^–1^.^6^ These batteries also offer rapid dynamic response times, typically in subsecond intervals, making them well-suited for applications that require both high power output and long-term reliability. However, remaining challenges include costs related to pressure containment, H_2_ permeation losses, and catalyst degradation, which continue to be actively addressed in the development of next-generation systems. Decoupled electrolyzers function at current densities of 0.1–1 A cm^–2^ ^17,25,38–40^ with Faradaic efficiencies near 100%^17^ and overall voltage efficiencies approaching 90%^23^ relative to the higher heating value of H_2_. Demonstrated lifetimes are 1000–10000 h, limited mainly by mediator stability. Their advantage lies in gas purity and safety; their challenge is achieving high areal throughput at a low capital cost.

To compare devices serving different functions, common descriptors are essential. Table 1 summarizes the main characteristics and performance metrics of the M–H_2_ batteries and decoupled electrolyzers discussed in this Perspective. Reporting geometric current density alongside specific current (A g^–1^) and full metadata (temperature, pH, pressure, flow, and separator details) enables reproducibility across laboratories. The table highlights a clear maturity gap: NiMH and Ni–H_2_ batteries have already reached the highest levels of technology-readiness level (TRL 9 and 8–9, respectively) and routinely undergo thousands to tens-of-thousands of cycles, while decoupled electrolyzers are still in the laboratory-to-pilot phase (TRL 3–6), with correspondingly shorter demonstrated durability tests. This difference in development stage means that, today, closed battery formats can be deployed with confidence where proven reliability is essential, while membrane-free electrolyzers remain at the proof-of-concept stage and will require further scale-up before they can be commercially viable. In addition, Table 1 further reveals that performance data are commonly reported under non-uniform testing protocols and with incomplete metadata (e.g., catalyst loading, normalization basis, pressure/flow conditions, and explicit efficiency definitions). A minimal, cross-field reporting package would improve comparability and hasten the translation of established durability methodologies and a mechanistic understanding across communities. Such harmonization transforms isolated demonstrations into a quantitative continuum: batteries prioritizing round-trip efficiency and cycle life, electrolyzers prioritizing Faradaic efficiency and scalability. The overlap region, where both store and generate H_2_ reversibly, is where innovation will matter most.

*Standardized, cross-field benchmarks—reporting geometric current density, mediator retention, gas purity, and the full H*_*2*_ *balance per cycle—are the quickest way to turn prototypes into commercially viable products.*

**Table 1:**
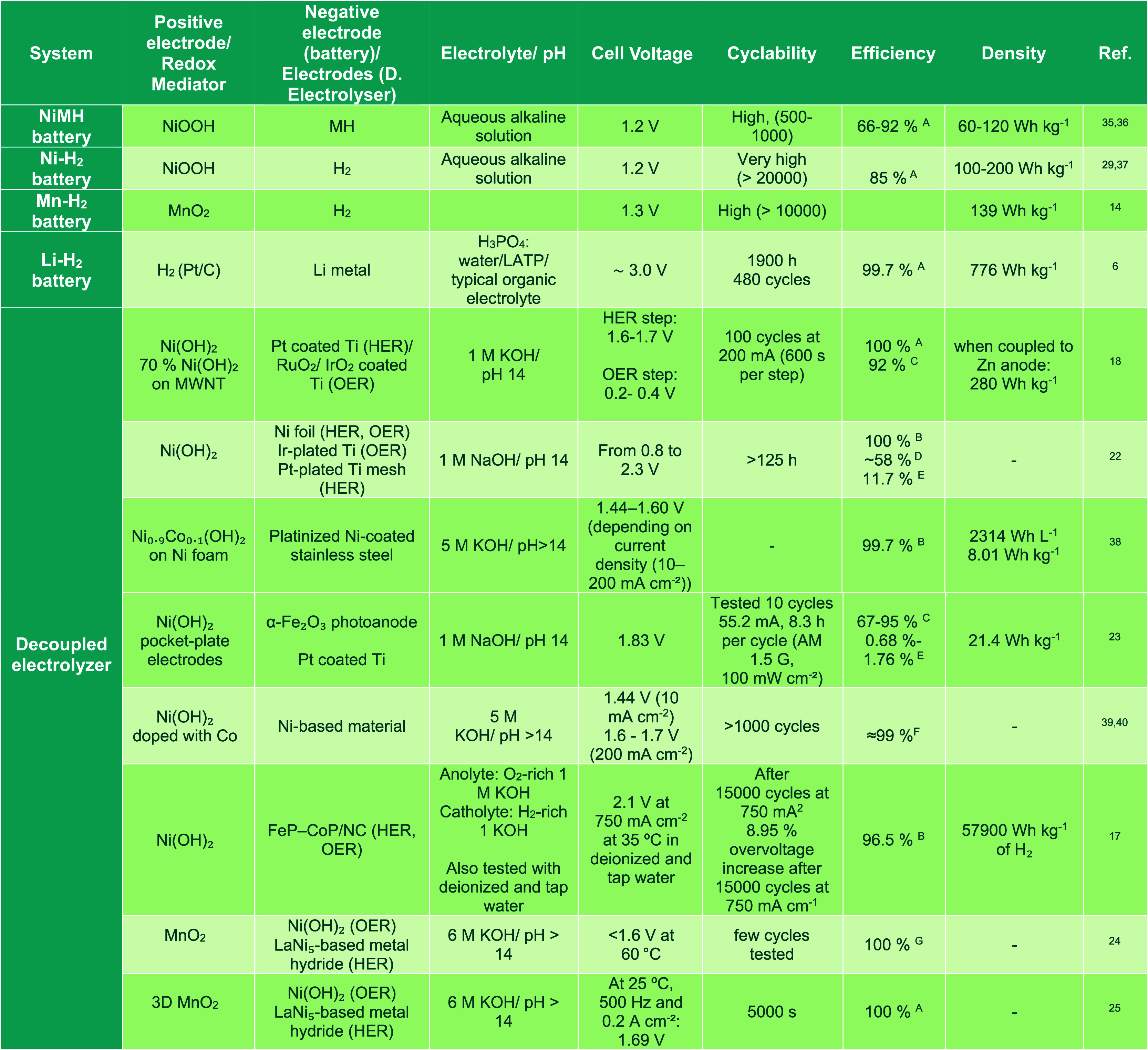
Key Characteristics and Performance Metrics of M–H_2_ Batteries and Decoupled Electrolyzers^a^ ^,^ ^a^

a Abbreviations: A, Coulombic efficiency; B, Faradaic efficiency; C, voltage efficiency; D, electrolysis efficiency; E, sun to hydrogen efficiency (STH); F, higher heating value of hydrogen (HHV); G, capacity efficiency.

b See References 6, 14, 17, 18, 22, 23, 24, 25,^29^,^35^,^36^,^37^,^38^,^39^, and^40^ .

To make the call for harmonized testing more actionable, Table 2 condenses a minimal set of cross-field benchmarks that can be adopted immediately by both the M–H_2_ battery and decoupled-electrolyzer communities. Rather than prescribing device-specific architectures, the table aligns the three critical functional blocks—mediator/electrode, H_2_ electrode, and separator or gas-management element—under a shared intermittent-load duty profile and a short list of universally reportable readouts (durability under cycling, polarization drift, and a closed H_2_ balance with purity/crossover checks). The intent is to provide a common “baseline test package” that enables reproducible, laboratory-to-laboratory comparisons and clarifies whether performance losses originate from mediator instability, H_2_-electrode degradation, or transport/sealing limitations.

**Table 2:**
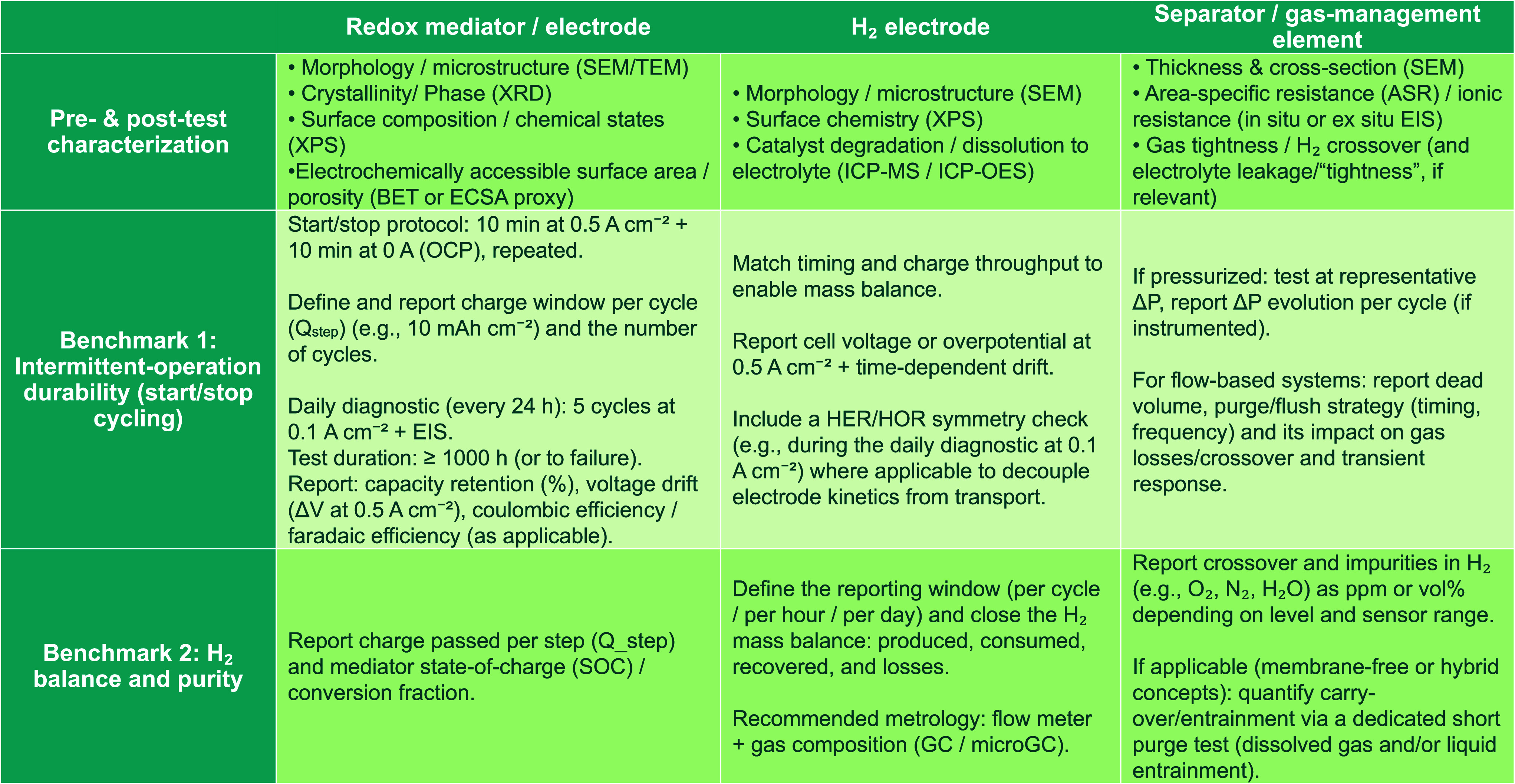
Proposed Standardized Protocols^a^

a Abbreviations: SEM, scanning electron microscope; TEM, transmission electron microscopy; XRD, X-ray diffraction; XPS, X-ray photoelectron spectroscopy; BET, surface area calculation using Brunauer–Emmett–Teller model; ECSA, electrochemically active surface area; ICP-MS, inductively coupled plasma–mass spectrometry; ICP-OES, inductively coupled plasma–optical emission spectroscopy; EIS, electrochemical impedance spectroscopy; Δ*P*, change in pressure; OCP, open circuit potential; Δ*V*, change in volume; HER, hydrogen evolution reaction; OER, oxygen evolution reaction; GC, gas chromatography; microGC, micro gas chromatography;

## Materials, Mechanisms, and Integration Opportunities

A unified view highlights three intertwined material challenges: (i) mediator durability, (ii) H_2_-electrode design, and (iii) interfacial management. Addressing them jointly can accelerate progress in both device types.

Nickel hydroxide (Ni­(OH)_2_) remains the benchmark for decoupled electrolyzers and M–H_2_ batteries due to its low cost, stability, and well-understood cycling behavior. Other promising materials, such as MnO_2_/MnOOH, Bi/Bi_2_O_3_, and Zn/Zn­(OH)_2_ further extend the electrochemical window by offering higher areal capacities and more robust cycling stability at different operational voltages. These materials enable broader application in energy systems that require flexible operating conditions. However, the next generation of mediators faces several challenges. First, capacity retention during prolonged cycling remains a critical issue.^41^ For materials to compete effectively in commercial applications, they must be capable of maintaining over 50 mAh cm^–2^ of reversible capacity at high current densities (≥0.5 A cm^–2^) with less than 10% polarization drift after 1000 h of operation. Achieving this will require further improvements in electrode design, material purity, and electrolyte management to minimize degradation during cycling. Furthermore, ensuring scalability and sustainability of synthesis methods using earth-abundant materials is essential for reducing costs and enabling large-scale deployment. Materials such as MnO_2_/MnOOH, Bi/Bi_2_O_3_, and Zn­(OH)_2_, though promising, still face challenges related to material synthesis, cost, and long-term stability in harsh electrochemical environments, particularly under variable operational conditions.

A recent innovative approach by Dotan et al.^38^ presents an alternative, multistep mechanism for water splitting, which introduces a novel integration of electrochemical and thermal activation to optimize hydrogen and oxygen production. This two-step process, as illustrated in Figure 3, first involves an electrochemical step for H_2_ evolution followed by a thermally activated chemical step for oxygen evolution, both of which are decoupled in time. The use of this dual mechanism could pave the way for more efficient, scalable, and energy-saving systems in water-splitting technologies. What distinguishes this approach is the second, thermally activated step. After electrochemical charging, the anode is removed from the electrochemical cell and immersed in a hot 5 M KOH solution at 95 °C, where it spontaneously regenerates, transitioning from Ni_0_._9_Co_0_._1_OOH back to Ni_0_._9_Co_0_._1_(OH)_2_, while releasing oxygen without the need for any external electrical potential. This thermal step reduces energy input and enhances the overall efficiency of the process. This method has been tested in both alkaline and carbonate–bicarbonate electrolytes, achieving solid performance even at lower pH levels (around pH 10.6). The scalability of this concept was also explored by the authors, who proposed a multicell prototype operating in swing mode. In this system, each cell has stationary anodes and cathodes, with only the electrolyte being cycled between the hydrogen and oxygen production stages. To prevent cross-contamination between the hydrogen- and oxygen-saturated electrolytes, an intermediate fluid is used to flush each cell between cycles, ensuring clean separation and efficiency. The use of thermal activation also opens new pathways for integrating waste heat recovery and optimizing the overall energy balance. The proposed swing-mode operation in a multicell system further enhances the potential for large-scale, continuous operations, which could prove critical in future H_2_ production plants that aim for cost reduction and sustainability.^38^

**Figure 3:**
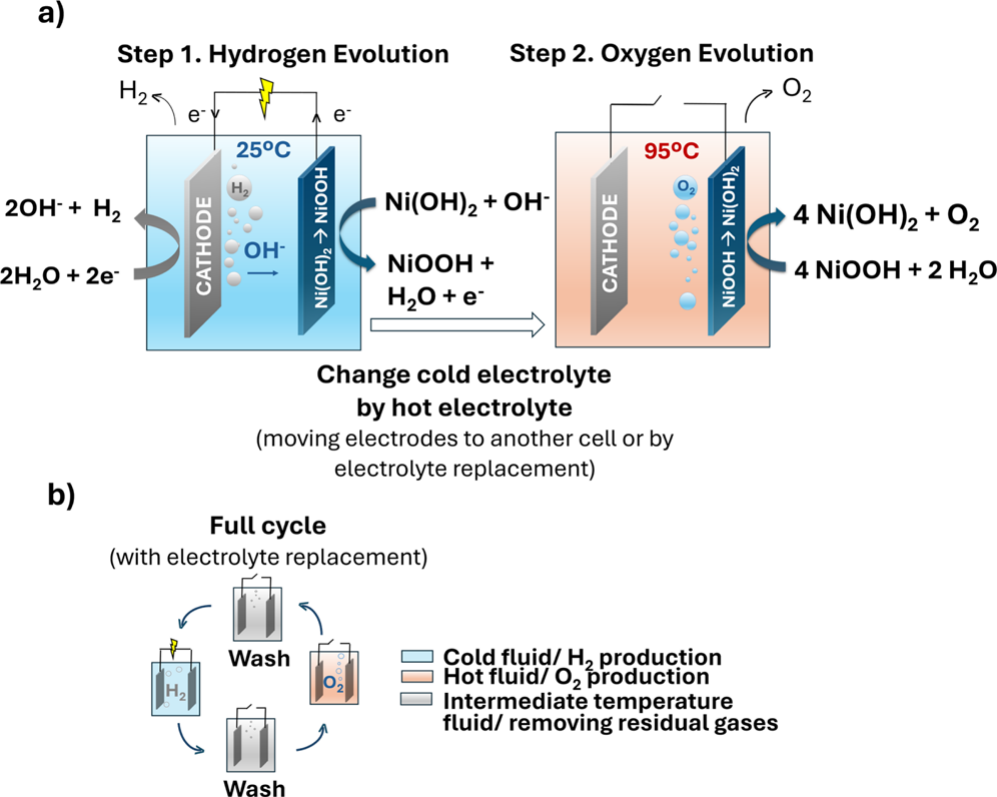
(a) Conceptual scheme of the electrochemical– thermochemical-activated approach proposed by Dotan et al.;^38^ (b) operating sequence of a full cycle proposed in the same work.

Replacing platinum-group catalysts with Ni–Mo–Co alloys in H_2_-storage electrodes has significantly reduced costs while maintaining the necessary kinetics for the HER/HOR. This substitution is crucial for improving the economic feasibility of M–H_2_ systems, particularly for large-scale and long-duration applications. However, while these alloys provide a viable alternative to precious metals, challenges persist in enhancing their stability, activity, and longevity over extended cycling. Ni–Mo–Co alloys are prone to corrosion and oxidation under the harsh conditions of long-term cycling, which can degrade their performance over time.^42^ Moreover, ensuring efficient H_2_ storage and reversible H_2_ desorption remains a key hurdle.^43^ Metal-hydride reservoirs such as LaNi_5_ or TiFe and moderate-pressure vessels offer safe, long-life H_2_ storage for stationary systems,^10^ while Li–H_2_ and Mn–H_2_ prototypes probe high-voltage frontiers.^6,14,15^ The capacity of H_2_-storage electrodes must be optimized to maintain both gravimetric and volumetric energy density, particularly for next-generation systems requiring high-rate performance and sustained cycling efficiency. Reporting the H_2_ balance per cycle and permeation losses with standardized methods will also be critical for technology comparisons.

Gas evolution and consumption in electrochemical systems impose cyclic mechanical stresses and create local concentration gradients, which can degrade the performance over time. To manage these stresses, advanced 3D foam structures,^44^ wettability-graded^45^ coatings, and microchanneled electrodes^46^ are being developed to improve bubble management and ensure consistent ionic access during high-rate operations. Alkaline KOH or NaOH remains the most widely used electrolyte for both M–H_2_ batteries and decoupled electrolyzers due to its high ionic conductivity and compatibility with most H_2_-storage materials. However, the formation of carbonates under exposure to atmospheric CO_2_ can steadily degrade performance by decreasing the ionic conductivity and interfering with reaction kinetics.^47^ As a result, monitoring the conductivity and pH drift should be considered best practices for system diagnostics, especially in long-term stability studies. In the future, polymer–ceramic hybrid electrolytes may enable neutral or mildly alkaline environments, improving performance while minimizing the risks associated with gas crossover and carbonate formation. Such advancements would help create more efficient and durable systems for H_2_ storage and electrolysis, driving the field closer to commercial-scale implementation.

Integration at the system level follows naturally. Fast, closed M–H_2_ batteries can buffer high-frequency renewable fluctuations, while slower, open decoupled electrolyzers handle sustained surpluses, storing H_2_ for later reconversion.

## Economic Analysis

The main benefit of adopting standardized manufacturing for shared components in metal–hydrogen batteries and decoupled electrolyzers (e.g., electrodes/redox mediators, catalyst coatings, separators, etc.) is the increase in production volume of these components. Higher volumes enable cost reductions through economies of scale, lowering the unit cost of each standardized component. This, in turn, decreases overall device manufacturing costs and ultimately the commercial price of both metal–hydrogen batteries and decoupled electrolyzers. Lower-cost devices would improve the economic competitiveness of these technologies and facilitate their broader deployment as energy-conversion and energy-storage solutions. To put this general argument into context, we briefly summarize the main cost drivers of each technology.

Ni–H_2_ batteries derive much of their cost from materials and containment. In the classical aerospace design, approximately half of the materials costs are attributed to the stainless-steel pressure vessel, which must withstand cycling at pressures of >80 bar and be machined to precise standards. The next largest cost components are high-purity nickel foam and β-NiOOH paste for the positive electrode, followed by PGM catalysts (Pt and Ru) on the H_2_ electrode and zirconia-based separators for ionic insulation. The switch from Pt to a Ni–Mo–Co alloy significantly reduced the cost of active materials, bringing recent prototypes for grid storage down to approximately $83 kW h^–1^ at stack level, with further reductions possible as vessel fabrication is standardized and noble metals are phased out.^29,48^ Commercial entrants such as EnerVenue have reported similar cost targets below $80 kW h^–1^ once their Kentucky gigafactory reaches full volume, as Ni, steel, and alkaline electrolyte are globally commoditized, minimizing exposure to Li or Co price volatility.^49^ In contrast, nickel–metal-hydride packs eliminate the need for pressure vessels but still carry the cost of rare-earth AB_5_ hydride alloys. Because of this, these packs are classified as “moderate” in cost, while the cost of Ni–H_2_ remains “high” until scale effects take hold. In both chemistries, balance-of-plant is relatively simple, without the need for thermal runaway mitigation, fire suppression or heating, ventilation, and air conditioning (HVAC), resulting in BOS typically adding only 15–20% to cell cost, compared to 30–40% for Li-ion batteries.

Capital expenditure (CAPEX) dominates life-cycle economics only when the battery’s exceptional lifespan is overlooked. A pressure-vessel stack amortized over 30000 cycles and three decades yields a levelized cost of storage (LCOS) close to a few cents per kWh-cycle.^29^ Operational expenditure (OPEX) is mostly limited to electrolyte top-ups every few years and periodic calibration of pressure sensors, with no reported need for life-long thermal management overhauls. As a result, total OPEX sits well under 1% of CAPEX per year, and recycling value from stainless steel and Ni helps offset decommissioning costs. The maintenance advantage becomes more apparent when comparing LCOS trajectories: while Li-ion’s LCOS rises sharply for discharge durations exceeding 8 h, techno-economic roadmaps for Ni–H_2_ show LCOS remaining below $0.05 kW h^–1^ even at 100 h durations, since the same vessel can accept more H_2_ without requiring new electrodes.^50,51^

In contrast, decoupled electrolyzers aim to reduce costs by eliminating the polymer membrane separator, which currently drives hardware prices $1200–1400 kW^–1^ for AEL and PEM stacks.^52^ Instead, they replace the membrane with a more affordable, sustainable, and durable solid redox mediator. A recent techno-economic assessment of a membrane-free flow electrolyzer using FeP–CoP bifunctional catalysts projected a H_2_ cost of $1.81 kg^–1^ at a 750 mA cm^–2^ operating point, comfortably below the market target of $2 kg^–1^. Roughly two-thirds of this cost is electricity, while stack CAPEX drops to less than $300 kW^–1^ when the separator and PGM catalyst are eliminated—an estimate consistent with academic analyses that forecast 30% savings vs PEM electrolyzers.^27,53,54^ OPEX is primarily governed by mediator stability and pump energy. Most solid mediators reported retain over 90% capacity after 1000 h, resulting in an annual replacement cost at a few cents per kg of H_2_. Additionally, the decoupled architecture separates voltage stress from gas purity, extending catalyst lifetimes compared with conventional alkaline stacks.

To compare the two technologies economically, H_2_ must be translated into electricity or vice versa. At 50% round-trip electrolyzer-to-fuel-cell efficiency, a H_2_ price of $1.81 kg^–1^ corresponds to approximately $0.09 kW h^–1^ of delivered electricity, assuming the higher heating value. A Ni–H_2_ battery charged from the same renewable source delivers electricity for about $0.01 kW h^–1^ per cycle, but requires internal storage. For durations under a day, the battery is more cost-effective due to its already sunk CAPEX and negligible OPEX; however, for durations exceeding 48 h, hydrogen’s ability to add storage at around $20 kg^–1^ outcompetes the cost of additional pressure vessels or extra battery stacks.^55–57^

## Outlook and Research Roadmap

The most immediate challenge, and opportunity, for both M–H_2_ batteries and decoupled electrolyzers lies in establishing the language of comparison, a common foundation for durability and standardization. At present, reported metrics vary widely, making comparison difficult and sometimes misleading. Creating shared benchmark protocols, similar in spirit to those employed by the U.S. Department of Energy (DOE) for fuel-cell stacks or the International Electrotechnical Convention (IEC) for secondary batteries, would provide the necessary framework for fair evaluation. These protocols should quantify mediator retention, catalyst degradation, and gas purity under realistic intermittent duty cycles that mimic the fluctuating nature of renewable power. Only through reproducible, cross-laboratory testing will claims of stability or efficiency acquire genuine meaning.

The discovery of new materials for M–H_2_ batteries and decoupled electrolyzers will increasingly rely on computational design. Virtual screening using density functional theory (DFT) and machine learning can rapidly identify redox couples with optimal potential windows and stability, significantly shortening the discovery-to-deployment timeline. Coupled with automated synthesis and electrochemical testing, this approach will accelerate the development of mediators and dopants designed for both high capacity and long life. Furthermore, predictive modeling of degradation processes such as phase disproportionation, catalyst leaching, and carbonate formation will enable proactive mitigation strategies before these materials reach practical applications.

Equally critical is the development of nonprecious metal catalysts that can withstand the harsh conditions of the alkaline H_2_ electrode environment. Future catalysts must feature self-passivating or self-healing layers to maintain their long-term stability and performance. Innovations in nanostructuring, core–shell design, and hierarchical structures offer potential solutions to achieve both durability and high catalytic activity. Progress will also depend on advancing the interfaces where gas, liquid, and solid phases meet. The microscopic dynamics of bubble nucleation, local pH gradients, and mechanical stresses are critical to macroscopic stability. Integrating operando visualization techniques will allow for real-time analysis of material behavior under operational conditions. These insights will inform the design of textured electrodes and graded wettability surfaces capable of maintaining stable operation at high current densities, essential for scaling both systems.

Convergence between M–H_2_ batteries and decoupled electrolyzers will ultimately be proven by hybrid pilots that combine their respective strengths. Kilowatt-scale systems that switch between closed, short-term buffering and open, long-term H_2_ production should report mode-resolved metrics—round-trip efficiency (buffering) and kWh kg^–1^ H_2_, Faradaic efficiency, and product purity (production)—alongside switching penalties (ramp time, downtime, purge/flush, and hold-up losses) and balance-of-system parasitics (pumping/valving power, electrolyte conditioning, compression/drying, maintenance intervals).^58^ These pilots also expose the trade-offs: added hardware and control complexity raise CAPEX/OPEX and failure risk, while frequent switching can drive transient cross-contamination and accelerated degradation—making it essential to separate mode aging from switching-induced wear. Mitigation is practical and testable: modular skid architectures that minimize dead volume, bounded operating windows with validated duty cycles (including explicit switching-stress tests), and inline sensing with supervisory control (conductivity/pH, dissolved gas, polarization drift) to reduce overheads and to quantify the levelized cost of storage (LCOS) and levelized cost of hydrogen (LCOH) under identical tariff and duty profiles. These pilots also face clear limitations: added hardware and controls increase CAPEX/OPEX and failure modes, while frequent switching can introduce transient cross-contamination and accelerate degradation, making the attribution between “mode aging” and “switching-induced wear” essential. Practical mitigation includes modular skid-based architectures that minimize dead volumes, bounded operating windows and validated duty profiles (including explicit switching-stress tests), and inline sensing with supervisory control (e.g., conductivity/pH, dissolved gas, polarization drift) to reduce overheads and quantify LCOS and LCOH under identical tariff and duty profiles.

As these elements mature together, M–H_2_ batteries and decoupled electrolyzers will evolve from parallel experiments into interoperable components of the same sustainable infrastructure technologies and will no longer compete for research attention but reinforce one another. The ultimate goal is an integrated H_2_-enabled storage ecosystem in which electrons and molecules (H_2_) interchange seamlessly, according to grid demand.

Figure 4 summarizes a short-, mid-, and long-term roadmap highlighting where M–H_2_ batteries and decoupled electrolyzers converge or diverge: short-term focuses on standardized metrics/protocols under intermittent operation, mid-term on materials and interface co-design with modeling and operando validation, and long-term on hybrid demonstrations and bankable deployment with robust LCOS/LCOH benchmarking and standards alignment.

**Figure 4:**
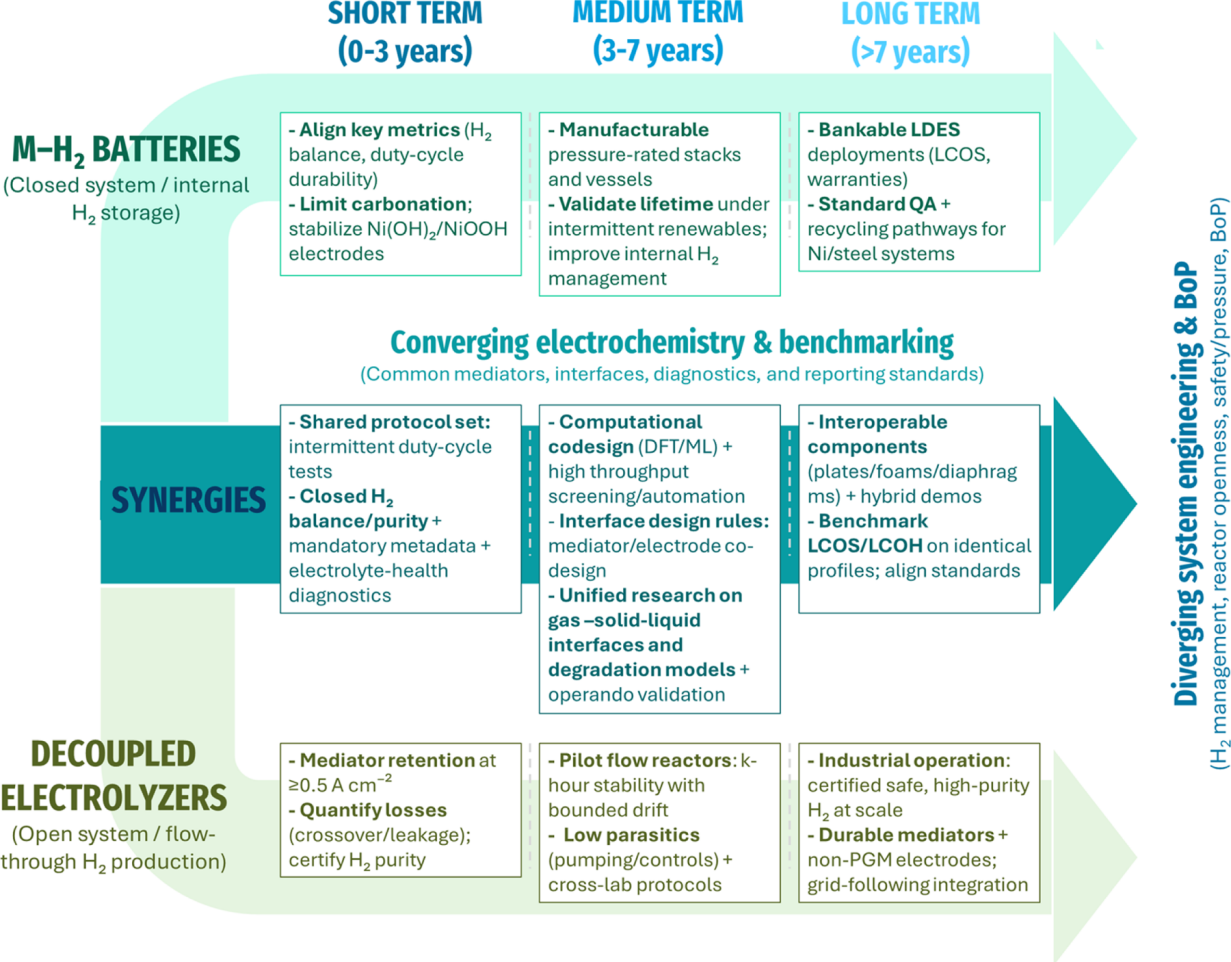
Roadmap highlighting convergence and divergence aspects of M–H_2_ batteries and decoupled electrolyzers.
